# (3*R*,6*S*,7a*S*)-3-Phenyl-6-(phenyl­sulfan­yl)perhydro­pyrrolo[1,2-*c*]oxazol-5-one

**DOI:** 10.1107/S1600536809011623

**Published:** 2009-04-02

**Authors:** Graeme J. Gainsford, Andreas Luxenburger, Anthony D. Woolhouse

**Affiliations:** a Industrial Research Limited, PO Box 31-310, Lower Hutt, New Zealand

## Abstract

Mol­ecules of the title compound  [systematic name: (2*R*,5*S*,7*S*)-2-phenyl-7-phenyl­sulfanyl-1-aza-3-oxa­bicyclo­[3.3.0]octan-8-one], C_18_H_17_NO_2_S, form high quality crystals even though they are only packed using C—H⋯O(carbon­yl) and weak C—H⋯S inter­actions. The dihedral angle between the aromatic rings is 85.53 (5)°. The fused rings adopt envelope and twist conformations.

## Related literature

For related structures, see Nagasaka & Imai (1995 ▶); Anwar *et al.* (2003 ▶); Bailey *et al.* (2000 ▶); McCarthy *et al.* (1999 ▶). For a description of the Cambridge Structural Database, see: Allen (2002 ▶).
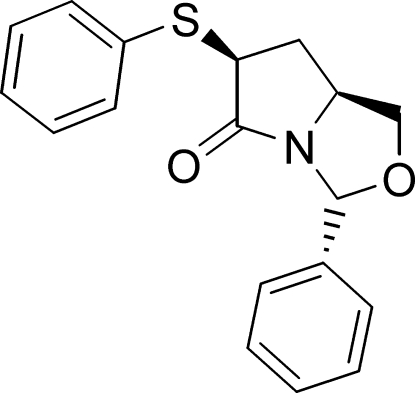


## Experimental

### 

#### Crystal data

C_18_H_17_NO_2_S
*M* *_r_* = 311.39
Orthorhombic, 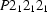

*a* = 5.3884 (3) Å
*b* = 11.2227 (7) Å
*c* = 25.3308 (16) Å
*V* = 1531.81 (16) Å^3^
*Z* = 4
Mo *K*α radiation
μ = 0.22 mm^−1^
*T* = 121 K
0.75 × 0.39 × 0.30 mm

#### Data collection

Bruker–Nonius APEXII CCD area-detector diffractometer
Absorption correction: multi-scan (Blessing, 1995 ▶) *T*_min_ = 0.829, *T*_max_ = 0.937
45370 measured reflections
5948 independent reflections
5711 reflections with *I* > 2σ(*I*)
*R*_int_ = 0.030

#### Refinement

*R*[*F* ^2^ > 2σ(*F* ^2^)] = 0.033
*wR*(*F* ^2^) = 0.087
*S* = 1.11
5948 reflections
199 parameters
H-atom parameters constrained
Δρ_max_ = 0.38 e Å^−3^
Δρ_min_ = −0.20 e Å^−3^
Absolute structure: Flack (1983 ▶), 2508 Friedel pairs
Flack parameter: 0.01 (4)

### 

Data collection: *APEX2* (Bruker, 2006 ▶); cell refinement: *SAINT* (Bruker, 2006 ▶); data reduction: *SAINT* and *SADABS* (Bruker, 2006 ▶); program(s) used to solve structure: *SHELXS97* (Sheldrick, 2008 ▶); program(s) used to refine structure: *SHELXL97* (Sheldrick, 2008 ▶); molecular graphics: *ORTEP-3* (Farrugia, 1997 ▶), *Mercury* (Macrae *et al.*, 2006 ▶) and *PLATON* (Spek, 2009 ▶); software used to prepare material for publication: *SHELXL97* and *PLATON*.

## Supplementary Material

Crystal structure: contains datablocks global, I. DOI: 10.1107/S1600536809011623/er2064sup1.cif

Structure factors: contains datablocks I. DOI: 10.1107/S1600536809011623/er2064Isup2.hkl

Additional supplementary materials:  crystallographic information; 3D view; checkCIF report

## Figures and Tables

**Table 1:** Hydrogen-bond geometry (Å, °)

*D*—H⋯*A*	*D*—H	H⋯*A*	*D*⋯*A*	*D*—H⋯*A*
C7*A*—H7*A*⋯O5^i^	1.00	2.55	3.4305 (13)	147
C7—H72⋯O5^ii^	0.99	2.62	3.3493 (16)	131
C1—H1*B*⋯O5^ii^	0.99	2.71	3.1513 (13)	108
C1—H1*A*⋯S1^ii^	0.99	3.00	3.9512 (14)	162
C4—H4⋯S1^iii^	0.95	2.98	3.7531 (13)	139

Symmetry codes: (i) 
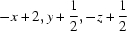
; (ii) 
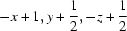
; (iii) 
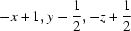
.

**Fig. 1.**
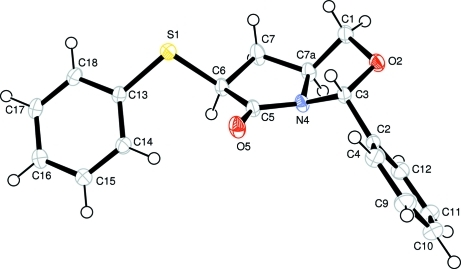
Molecular structure of the asymmetric unit (Farrugia, 1997); displacement ellipsoids are shown at the 50% probability level.

**Fig. 2.**
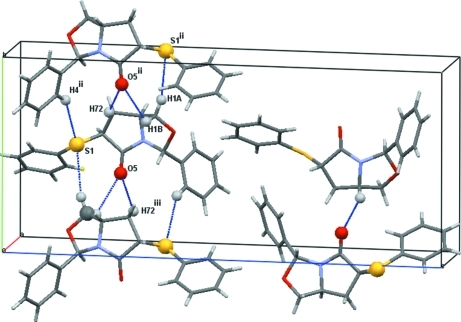
Packing diagram (Mercury; Macrae et al.,2006) of the unit cell. Contact atoms are shown as balls; not all interactions and labels are shown for clarity (see Table 1).

**Fig. 3.**
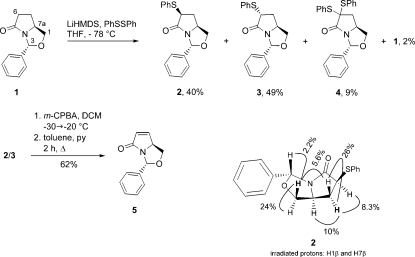
Preparation of the title compound.

## supplementary crystallographic information

### Comment

During the course of our efforts to delineate, in detail, the conversion of the bicyclic lactam 1 into its highly prized, chiral α,β-unsaturated derivative 5 employing *sulfur chemistry* (Fig. 3), we were able to isolate and separate the three anticipated products (2,3 & 4) from the rapid quenching of the lithium enolate of 1 with the electrophile, PhSSPh: the two 6-(phenylthio)-mono-adducts 2 and 3 were found to be predominant in the mixture with up to 10% only of the 6,6-bis(phenylthio)-adduct. In order to be able to unequivocally assign structures to each of the mono-adducts, we carried out an X-ray crystallographic study of the title *cis*(*endo*-) adduct 2 (Scheme) so that structural inferences made on the basis of nOe experiments could be corroborated (see bottom right entry in fig. 3).

The 2-(*p*-methoxyphenyl) derivative is described (molecule 21*b*) by Nagasaka & Imai (1995). Related compounds in the Cambridge Structural Database [C.S.D. Version 5.30 with November 2008 updates (Allen, 2002)] have been reported by Anwar *et al.* (2003) (IJAGUV) and Bailey *et al.* (2000) (QINMEF & QINMEJ).

The asymmetric unit is shown in Figure 1. The 5-membered fused rings (N4,C5,C6,C7,C7a) and (C1,O2,C3,N4,C7a) are best described as having envelope (on C6) and twist (on C1—O2) conformations (Spek, 2009). The 3-phenyl (C2,C4,C9—C12) and 6-phenylthio (C13—C18) phenyl rings subtend angles of 85.53 (5)° to each other and 71.74 (5) and 39.74 (5)° respectively to the mean plane through the fused ring (octan-8-one) atoms. The corresponding three angles for the 6-benzyl closest structural relative (QINMEF) are 80.14 (7), 71.18 (6) and 36.82 (7)° while the interplanar angles found for the 3-phenyl rings in IJAGUV & QINMIJ structures are 74.1 (4) and 76.73 (7)%, respectively.

The molecular packing is provided by mainly weak C–H···O interactions (entries 1–3, Table 1, involving a bifurcated O) as partly shown in Figure 2. The weak, just significant, C—H···S interactions have been observed before contributing to dimer formation in CUQXUH with a C—H···S angles of 140° (McCarthy *et al.*, 1999).

These crystals were of superb quality, confirmed by the final agreement indices and difference density maps, which raises the issue of how many of the weak intermolecular interactions may just be fortuitous, with packing largely determined by the overall molecular shape and van der Waal's forces.

### Experimental

To a solution of the lactam 1 (19.7 g, 97.0 mmol) in dry THF (180 ml) was added dropwise LiHMDS (1 *M* solution in hexanes; 116 ml, 116 mmol) at -78 °C and the solution was stirred for further 60 min before a concentrated solution of diphenylsulfide (25.4 g, 116 mmol) in dry THF (25 ml) was added rapidly in one single portion at the same temperature. After being stirred for 3 h the reaction mixture was quenched at -78 °C with saturated aqueous ammonium chloride solution (50 ml) and allowed to warm to ambient temperature before being diluted with ethyl acetate (400 ml). The separated organic phase was washed with water and brine, dried over MgSO_4_ and concentrated to give a crude oil, the composition of which was determined by HPLC analysis. Based on this analysis the two diastereomeric phenylthioethers 2 and 3 were formed in 40% and 49% yield, respectively. In addition, the reaction provided the bis(phenylthio)-adduct 4 in 9% as the by-product and 2% of the starting material remained unreacted. The crude product was finally fractionated by flash column chromatography (silica gel, 10% and 20% ethyl acetate/petroleum ether) to yield the three adducts 2, 3 and 4.

(2): (3*R*,6*S*,7a*S*)-3-Phenyl-6-(phenylthio)tetrahydropyrrolo [1,2-*c*]oxazol-5(1*H*)-one was recrystallized from ethyl acetate/petroleum ether to give colourless crystals, *R*_f_ = 0.44 (30% ethyl acetate/petroleum ether); m.p. 97–98 °C, [α]^21^_D_=+215.6 (*c* 0.545, CHCl_3_); FTIR (neat) 1692 (C=O) cm^-1^; ^1^H NMR (500 MHz, CDCl_3_) δ 7.57–7.53 (m, 2H), 7.46–7.42 (m, 2H), 7.38–7.27 (m, 6H), 6.29 (s, 1H, H3), 4.26 (t, J=9.7 Hz, 1H, H6), 4.17 (dd, J=8.2, 6.2 Hz, 1H, H1α), 4.06–3.99 (m, 1H, H7aα), 3.21 (t, J=8.1 Hz, 1H, H1β), 2.81 (ddd, J=13.5, 9.2, 7.3 Hz, 1H, H7α), 1.93 (ddd, J=13.5, 10.2, 6.4 Hz, 1H, H7β); ^13^C NMR (125 MHz, CDCl_3_) δ 173.54, 138.18, 132.91, 132.85, 128.94, 128.55, 128.29, 127.89, 125.82, 87.18, 71.88, 55.73, 51.06, 32.03; HRMS (ES+) m/*z* calcd for C_18_H_17_NO_2_SNa^+^ 334.0878, obsd 334.0872; Anal. calcd for C_18_H_17_NO_2_S: C, 69.43; H, 5.50; N, 4.50. Found: C, 69.32; H, 5.63; N, 4.63.

(3): (3*R*,6*R*,7a*S*)-3-Phenyl-6-(phenylthio)tetrahydropyrrolo [1,2-*c*]oxazol-5(1*H*)-one, colourless crystals, *R*_f_ = 0.30 (30% ethyl acetate/petroleum ether); m.p. 80 °C; [α]^20^_D_ = +102.3 (*c* 1.05, CHCl_3_); FTIR (neat) 1702 (C=O) cm^-1^; ^1^H NMR (500 MHz, DMSO-*d_6_*) δ 7.52–7.48 (m, 2H); 7.41–7.30 (m, 6H); 7.27–7.23 (m, 2H), 6.05 (s, 1H, H3); 4.22 (dd, J=9.4, 3.3 Hz, 1H, H6); 4.13 (dd, J=8.0, 6.2 Hz, 1H, H1α); 3.90–3.83 (m, 1H, H7aα); 3.47 (t, J=8.4 Hz, 1H, H1β); 2.56 (ddd, J=14.5, 9.2, 5.3 Hz, 1H, H7β); 2.28 (ddd, J=14.2, 7.5, 3.3, 1H, H7α); ^13^C NMR (125 MHz, DMSO-*d_6_*) δ 174.58, 138.57, 132.55, 131.99, 129.02, 128.49, 128.25, 127.76, 125.93, 86.47, 70.97, 57.11, 49.25, 30.66; HRMS (ES+) m/*z* calcd for C_18_H_17_NO_2_SNa^+^ 334.0878, obsd 334.0879; Anal. calcd for C_18_H_17_NO_2_S: C, 69.43; H, 5.50; N, 4.50. Found C, 69.39; H, 5.59; N, 4.55.

(4): (3*R*,7a*S*)-3-Phenyl-6,6- bis(phenylthio)tetrahydropyrrolo[1,2-*c*]oxazol-5(1*H*)-one, colourless crystals, *R*_f_ = 0.54 (30% ethyl acetate/petroleum ether); m.p. 108 °C; [α]^20^_D_ = +157.1 (*c* 0.62, CDCl_3_); FTIR (neat) 1698 (C=O) cm^-1^; ^1^H NMR (500 MHz, CDCl_3_) δ 7.71–7.68 (m, 2H), 7.59–7.55 (m, 2H), 7.41–7.29 (m, 9H), 7.25–7.20 (m, 2H), 6.19 (s, 1H), 4.03 (dd, J=8.1, 6.3 Hz, 1H), 3.69–3.63 (m, 1H), 3.09 (t, J=8.1 Hz, 1H), 2.54 (dd, J=14.3, 7.0 Hz, 1H), 2.35 (dd, J=14.3, 6.5 Hz, 1H); ^13^C NMR (125 MHz, CDCl_3_) δ 171.71, 137.95, 136.65, 136.27, 131.18, 130.44, 129.96, 129.57, 129.01, 128.87, 128.67, 128.35, 126.02, 87.09, 71.78, 68.68, 55.00, 39.79; HRMS (ES+) m/*z* calcd for C_24_H_21_NO_2_S_2_Na^+^ 442.0911, obsd 442.0191; Anal. calcd for C_24_H_21_NO_2_S_2_: C, 68.70; H, 5.04; N, 3.34. Found C, 68.88; H, 5.29; N, 3.37.

### Refinement

A total of 9 reflections within 2θ 68° were omitted from refinement as either outliers or partially screened by the backstop. All H atoms bound to carbon were constrained to their expected geometries (C—H 0.95, 0.99 & 1.00 Å). All H atoms were refined with *U*_iso_ 1.2 times that of the *U*_eq_ of their parent atom.

### Crystal data

**Table:**

C_18_H_17_NO_2_S	*F*(000) = 656
*M**_r_* = 311.39	*D*_x_ = 1.350 Mg m^−^^3^
Orthorhombic, *P*2_1_2_1_2_1_	Mo *K*α radiation, λ = 0.71073 Å
Hall symbol: P 2ac 2ab	Cell parameters from 9806 reflections
*a* = 5.3884 (3) Å	θ = 3.0–34.4°
*b* = 11.2227 (7) Å	µ = 0.22 mm^−^^1^
*c* = 25.3308 (16) Å	*T* = 121 K
*V* = 1531.81 (16) Å^3^	Block, colourless
*Z* = 4	0.75 × 0.39 × 0.30 mm

### Data collection

**Table:**

Bruker–Nonius APEXII CCD area-detector diffractometer	5948 independent reflections
Radiation source: fine-focus sealed tube	5711 reflections with *I* > 2σ(*I*)
graphite	*R*_int_ = 0.030
Detector resolution: 8.333 pixels mm^-1^	θ_max_ = 34.0°, θ_min_ = 3.0°
φ and ω scans	*h* = −8→8
Absorption correction: multi-scan (Blessing, 1995)	*k* = −17→16
*T*_min_ = 0.829, *T*_max_ = 0.937	*l* = −38→38
45370 measured reflections	

### Refinement

**Table:**

Refinement on *F*^2^	Secondary atom site location: difference Fourier map
Least-squares matrix: full	Hydrogen site location: inferred from neighbouring sites
*R*[*F*^2^ > 2σ(*F*^2^)] = 0.033	H-atom parameters constrained
*wR*(*F*^2^) = 0.087	*w* = 1/[σ^2^(*F*_o_^2^) + (0.0528*P*)^2^ + 0.1721*P*] where *P* = (*F*_o_^2^ + 2*F*_c_^2^)/3
*S* = 1.11	(Δ/σ)_max_ = 0.001
5948 reflections	Δρ_max_ = 0.38 e Å^−^^3^
199 parameters	Δρ_min_ = −0.20 e Å^−^^3^
0 restraints	Absolute structure: Flack (1983), 2508 Friedel pairs
Primary atom site location: structure-invariant direct methods	Flack parameter: 0.01 (4)

### Special details

**Table:**

Geometry. All e.s.d.'s (except the e.s.d. in the dihedral angle between two l.s. planes) are estimated using the full covariance matrix. The cell e.s.d.'s are taken into account individually in the estimation of e.s.d.'s in distances, angles and torsion angles; correlations between e.s.d.'s in cell parameters are only used when they are defined by crystal symmetry. An approximate (isotropic) treatment of cell e.s.d.'s is used for estimating e.s.d.'s involving l.s. planes.
Refinement. Refinement of *F*^2^ against ALL reflections. The weighted *R*-factor *wR* and goodness of fit *S* are based on *F*^2^, conventional *R*-factors *R* are based on *F*, with *F* set to zero for negative *F*^2^. The threshold expression of *F*^2^ > σ(*F*^2^) is used only for calculating *R*-factors(gt) *etc*. and is not relevant to the choice of reflections for refinement. *R*-factors based on *F*^2^ are statistically about twice as large as those based on *F*, and *R*- factors based on ALL data will be even larger.

### Fractional atomic coordinates and isotropic or equivalent isotropic displacement parameters (Å^2^)

**Table:**

	*x*	*y*	*z*	*U*_iso_*/*U*_eq_	
S1	0.53402 (5)	0.52168 (2)	0.147539 (9)	0.02284 (6)	
O2	0.63842 (15)	0.59460 (6)	0.36457 (3)	0.02259 (14)	
O5	0.76332 (18)	0.36178 (6)	0.23809 (3)	0.02532 (15)	
N4	0.80755 (16)	0.53105 (7)	0.28760 (3)	0.01873 (14)	
C1	0.5697 (2)	0.67901 (8)	0.32437 (4)	0.02362 (18)	
H1A	0.5735	0.7616	0.3381	0.028*	
H1B	0.4020	0.6619	0.3103	0.028*	
C2	0.88109 (18)	0.41716 (8)	0.37229 (3)	0.01718 (15)	
C3	0.70664 (17)	0.48760 (8)	0.33825 (3)	0.01756 (15)	
H3	0.5543	0.4392	0.3313	0.021*	
C4	0.8234 (2)	0.29955 (8)	0.38433 (4)	0.02221 (17)	
H4	0.6793	0.2640	0.3695	0.027*	
C5	0.77799 (18)	0.46990 (8)	0.24169 (3)	0.01866 (15)	
C6	0.7641 (2)	0.56099 (8)	0.19659 (4)	0.02006 (16)	
H6	0.9305	0.5691	0.1795	0.024*	
C7	0.6940 (3)	0.67833 (8)	0.22432 (4)	0.0274 (2)	
H71	0.7857	0.7462	0.2087	0.033*	
H72	0.5138	0.6938	0.2212	0.033*	
C7A	0.7678 (2)	0.66023 (8)	0.28236 (4)	0.02122 (17)	
H7A	0.9223	0.7055	0.2911	0.025*	
C9	0.9747 (2)	0.23345 (9)	0.41785 (5)	0.02726 (19)	
H9	0.9343	0.1531	0.4259	0.033*	
C10	1.1843 (2)	0.28528 (10)	0.43943 (4)	0.0288 (2)	
H10	1.2878	0.2406	0.4624	0.035*	
C11	1.2439 (2)	0.40310 (10)	0.42745 (5)	0.0274 (2)	
H11	1.3877	0.4386	0.4424	0.033*	
C12	1.09340 (18)	0.46886 (9)	0.39371 (4)	0.02237 (17)	
H12	1.1353	0.5489	0.3853	0.027*	
C13	0.71008 (18)	0.45302 (7)	0.09728 (4)	0.01856 (15)	
C14	0.9116 (2)	0.37876 (9)	0.10770 (4)	0.02280 (18)	
H14	0.9565	0.3611	0.1431	0.027*	
C15	1.0467 (2)	0.33070 (10)	0.06609 (4)	0.02566 (19)	
H15	1.1859	0.2813	0.0732	0.031*	
C16	0.9801 (2)	0.35426 (9)	0.01414 (4)	0.02609 (19)	
H16	1.0746	0.3221	−0.0142	0.031*	
C17	0.7743 (2)	0.42516 (9)	0.00390 (4)	0.02471 (18)	
H17	0.7254	0.4397	−0.0316	0.030*	
C18	0.63983 (19)	0.47488 (9)	0.04504 (4)	0.02167 (16)	
H18	0.5001	0.5238	0.0377	0.026*	

### Atomic displacement parameters (Å^2^)

**Table:**

	*U*^11^	*U*^22^	*U*^33^	*U*^12^	*U*^13^	*U*^23^
S1	0.02433 (10)	0.02516 (10)	0.01902 (10)	0.00568 (8)	−0.00096 (8)	−0.00069 (8)
O2	0.0304 (3)	0.0205 (3)	0.0169 (3)	0.0075 (3)	0.0009 (3)	−0.0014 (2)
O5	0.0404 (4)	0.0151 (3)	0.0205 (3)	0.0020 (3)	−0.0005 (3)	−0.0027 (2)
N4	0.0270 (4)	0.0144 (3)	0.0148 (3)	0.0010 (3)	0.0016 (3)	−0.0007 (2)
C1	0.0323 (5)	0.0203 (4)	0.0183 (4)	0.0070 (3)	−0.0006 (3)	−0.0002 (3)
C2	0.0203 (4)	0.0172 (3)	0.0141 (3)	0.0012 (3)	0.0009 (3)	−0.0011 (2)
C3	0.0218 (4)	0.0166 (3)	0.0143 (3)	0.0006 (3)	0.0007 (3)	−0.0006 (2)
C4	0.0250 (4)	0.0179 (3)	0.0236 (4)	−0.0004 (3)	−0.0009 (3)	−0.0001 (3)
C5	0.0243 (4)	0.0167 (3)	0.0150 (3)	0.0018 (3)	0.0006 (3)	−0.0009 (3)
C6	0.0282 (4)	0.0173 (3)	0.0147 (4)	0.0012 (3)	0.0007 (3)	0.0002 (3)
C7	0.0481 (6)	0.0161 (3)	0.0180 (4)	0.0049 (4)	0.0023 (4)	0.0002 (3)
C7A	0.0305 (5)	0.0140 (3)	0.0191 (4)	−0.0005 (3)	0.0014 (3)	−0.0016 (3)
C9	0.0311 (5)	0.0220 (4)	0.0287 (5)	0.0036 (4)	0.0001 (4)	0.0047 (3)
C10	0.0285 (5)	0.0328 (5)	0.0251 (5)	0.0093 (4)	−0.0033 (4)	0.0021 (4)
C11	0.0213 (4)	0.0334 (5)	0.0276 (5)	0.0029 (4)	−0.0047 (4)	−0.0034 (4)
C12	0.0197 (4)	0.0229 (4)	0.0245 (4)	−0.0016 (3)	0.0002 (3)	−0.0016 (3)
C13	0.0228 (4)	0.0169 (3)	0.0160 (3)	−0.0008 (3)	−0.0019 (3)	0.0000 (3)
C14	0.0280 (5)	0.0238 (4)	0.0167 (4)	0.0053 (3)	−0.0025 (3)	0.0008 (3)
C15	0.0268 (4)	0.0281 (4)	0.0222 (4)	0.0062 (4)	−0.0019 (4)	−0.0027 (3)
C16	0.0301 (5)	0.0293 (4)	0.0189 (4)	0.0002 (4)	0.0015 (4)	−0.0029 (3)
C17	0.0311 (5)	0.0272 (4)	0.0158 (4)	−0.0028 (4)	−0.0047 (4)	0.0004 (3)
C18	0.0251 (4)	0.0216 (4)	0.0183 (4)	−0.0007 (3)	−0.0053 (3)	0.0022 (3)

### Geometric parameters (Å, °)

**Table:**

S1—C13	1.7648 (10)	C7—H71	0.9900
S1—C6	1.8098 (10)	C7—H72	0.9900
O2—C3	1.4217 (11)	C7A—H7A	1.0000
O2—C1	1.4393 (12)	C9—C10	1.3831 (17)
O5—C5	1.2194 (11)	C9—H9	0.9500
N4—C5	1.3598 (11)	C10—C11	1.3940 (17)
N4—C7A	1.4716 (11)	C10—H10	0.9500
N4—C3	1.4762 (11)	C11—C12	1.3901 (15)
C1—C7A	1.5218 (15)	C11—H11	0.9500
C1—H1A	0.9900	C12—H12	0.9500
C1—H1B	0.9900	C13—C14	1.3942 (13)
C2—C4	1.3900 (12)	C13—C18	1.3981 (12)
C2—C12	1.3927 (13)	C14—C15	1.3899 (14)
C2—C3	1.5007 (13)	C14—H14	0.9500
C3—H3	1.0000	C15—C16	1.3896 (14)
C4—C9	1.3913 (15)	C15—H15	0.9500
C4—H4	0.9500	C16—C17	1.3892 (16)
C5—C6	1.5348 (13)	C16—H16	0.9500
C6—C7	1.5395 (13)	C17—C18	1.3866 (15)
C6—H6	1.0000	C17—H17	0.9500
C7—C7A	1.5363 (14)	C18—H18	0.9500
			
C13—S1—C6	103.50 (5)	H71—C7—H72	108.8
C3—O2—C1	106.90 (7)	N4—C7A—C1	100.10 (8)
C5—N4—C7A	113.75 (8)	N4—C7A—C7	104.74 (7)
C5—N4—C3	122.24 (8)	C1—C7A—C7	118.00 (10)
C7A—N4—C3	110.50 (7)	N4—C7A—H7A	111.1
O2—C1—C7A	102.90 (8)	C1—C7A—H7A	111.1
O2—C1—H1A	111.2	C7—C7A—H7A	111.1
C7A—C1—H1A	111.2	C10—C9—C4	119.71 (10)
O2—C1—H1B	111.2	C10—C9—H9	120.1
C7A—C1—H1B	111.2	C4—C9—H9	120.1
H1A—C1—H1B	109.1	C9—C10—C11	120.05 (10)
C4—C2—C12	119.60 (9)	C9—C10—H10	120.0
C4—C2—C3	119.09 (9)	C11—C10—H10	120.0
C12—C2—C3	121.26 (8)	C12—C11—C10	120.22 (10)
O2—C3—N4	102.93 (7)	C12—C11—H11	119.9
O2—C3—C2	109.72 (7)	C10—C11—H11	119.9
N4—C3—C2	116.27 (8)	C11—C12—C2	119.82 (9)
O2—C3—H3	109.2	C11—C12—H12	120.1
N4—C3—H3	109.2	C2—C12—H12	120.1
C2—C3—H3	109.2	C14—C13—C18	119.67 (9)
C2—C4—C9	120.59 (10)	C14—C13—S1	122.90 (7)
C2—C4—H4	119.7	C18—C13—S1	117.43 (7)
C9—C4—H4	119.7	C15—C14—C13	119.76 (9)
O5—C5—N4	125.01 (9)	C15—C14—H14	120.1
O5—C5—C6	127.15 (8)	C13—C14—H14	120.1
N4—C5—C6	107.84 (7)	C16—C15—C14	120.60 (10)
C5—C6—C7	104.01 (7)	C16—C15—H15	119.7
C5—C6—S1	112.46 (6)	C14—C15—H15	119.7
C7—C6—S1	110.72 (8)	C17—C16—C15	119.47 (10)
C5—C6—H6	109.8	C17—C16—H16	120.3
C7—C6—H6	109.8	C15—C16—H16	120.3
S1—C6—H6	109.8	C18—C17—C16	120.49 (9)
C7A—C7—C6	105.07 (8)	C18—C17—H17	119.8
C7A—C7—H71	110.7	C16—C17—H17	119.8
C6—C7—H71	110.7	C17—C18—C13	119.95 (9)
C7A—C7—H72	110.7	C17—C18—H18	120.0
C6—C7—H72	110.7	C13—C18—H18	120.0
			
C3—O2—C1—C7A	−42.19 (10)	C5—N4—C7A—C1	124.87 (9)
C1—O2—C3—N4	30.62 (10)	C3—N4—C7A—C1	−17.02 (10)
C1—O2—C3—C2	155.00 (8)	C5—N4—C7A—C7	2.19 (12)
C5—N4—C3—O2	−145.28 (9)	C3—N4—C7A—C7	−139.70 (9)
C7A—N4—C3—O2	−7.18 (10)	O2—C1—C7A—N4	34.70 (9)
C5—N4—C3—C2	94.76 (11)	O2—C1—C7A—C7	147.49 (8)
C7A—N4—C3—C2	−127.14 (9)	C6—C7—C7A—N4	−14.67 (12)
C4—C2—C3—O2	125.46 (9)	C6—C7—C7A—C1	−124.87 (9)
C12—C2—C3—O2	−51.84 (11)	C2—C4—C9—C10	0.09 (16)
C4—C2—C3—N4	−118.31 (9)	C4—C9—C10—C11	−0.26 (17)
C12—C2—C3—N4	64.39 (11)	C9—C10—C11—C12	−0.13 (17)
C12—C2—C4—C9	0.47 (15)	C10—C11—C12—C2	0.70 (16)
C3—C2—C4—C9	−176.88 (9)	C4—C2—C12—C11	−0.86 (14)
C7A—N4—C5—O5	−167.92 (11)	C3—C2—C12—C11	176.43 (9)
C3—N4—C5—O5	−31.03 (16)	C6—S1—C13—C14	37.85 (9)
C7A—N4—C5—C6	11.47 (12)	C6—S1—C13—C18	−142.53 (7)
C3—N4—C5—C6	148.36 (8)	C18—C13—C14—C15	2.42 (15)
O5—C5—C6—C7	159.26 (12)	S1—C13—C14—C15	−177.97 (8)
N4—C5—C6—C7	−20.11 (11)	C13—C14—C15—C16	−1.15 (17)
O5—C5—C6—S1	39.41 (14)	C14—C15—C16—C17	−0.94 (17)
N4—C5—C6—S1	−139.96 (7)	C15—C16—C17—C18	1.75 (16)
C13—S1—C6—C5	−98.36 (7)	C16—C17—C18—C13	−0.48 (15)
C13—S1—C6—C7	145.76 (7)	C14—C13—C18—C17	−1.62 (14)
C5—C6—C7—C7A	20.79 (12)	S1—C13—C18—C17	178.75 (8)
S1—C6—C7—C7A	141.81 (8)		

### Hydrogen-bond geometry (Å, °)

**Table:**

*D*—H···*A*	*D*—H	H···*A*	*D*···*A*	*D*—H···*A*
C7A—H7A···O5^i^	1.00	2.55	3.4305 (13)	147
C7—H72···O5^ii^	0.99	2.62	3.3493 (16)	131
C1—H1B···O5^ii^	0.99	2.71	3.1513 (13)	108
C1—H1A···S1^ii^	0.99	3.00	3.9512 (14)	162
C4—H4···S1^iii^	0.95	2.98	3.7531 (13)	139

Symmetry codes: (i) −*x*+2, *y*+1/2, −*z*+1/2; (ii) −*x*+1, *y*+1/2, −*z*+1/2; (iii) −*x*+1, *y*−1/2, −*z*+1/2.

## References

[bb1] Allen, F. H. (2002). *Acta Cryst.* B**58**, 380–388. 10.1107/s0108768102003890 12037359

[bb2] Anwar, M., Bailey, J. H., Dickinson, L. C., Edwards, H. J., Goswami, R. & Moloney, M. G. (2003). *Org. Biomol Chem.* **1**, 2364–2376. 10.1039/b303924b 12945710

[bb3] Bailey, J. H., Byfield, A. T. J., Davis, P. J., Foster, A. C., Leech, M., Moloney, M. G., Muller, M. & Prout, C. K. (2000). *J. Chem. Soc. Perkin Trans. 1*, pp. 1977–1982.

[bb4] Blessing, R. H. (1995). *Acta Cryst.* A**51**, 33–38. 10.1107/s0108767394005726 7702794

[bb5] Bruker (2006). *APEX2*, *SAINT* and *SADABS* Bruker AXS Inc., Madison, Wisconsin, USA.

[bb6] Farrugia, L. J. (1997). *J. Appl. Cryst.* **30**, 565.

[bb7] Flack, H. D. (1983). *Acta Cryst.* A**39**, 876–881.

[bb8] Macrae, C. F., Edgington, P. R., McCabe, P., Pidcock, E., Shields, G. P., Taylor, R., Towler, M. & van de Streek, J. (2006). *J. Appl. Cryst.* **39**, 453–457.

[bb9] McCarthy, D. G., Collins, C. C., O’Driscoll, J. P. & Lawrence, S. E. (1999). *J. Chem. Soc. Perkin Trans. 1*, pp. 3667–3675.

[bb10] Nagasaka, T. & Imai, T. (1995). *Chem. Pharm. Bull* **43**, 1081–1088.

[bb11] Sheldrick, G. M. (2008). *Acta Cryst.* A**64**, 112–122. 10.1107/S0108767307043930 18156677

[bb12] Spek, A. L. (2009). *Acta Cryst.* D**65**, 148–155. 10.1107/S090744490804362X PMC2631630 19171970

